# Socioeconomic position, built environment and physical activity among children and adolescents: a systematic review of mediating and moderating effects

**DOI:** 10.1186/s12966-022-01385-y

**Published:** 2022-12-12

**Authors:** Oddbjørn Klomsten Andersen, Mekdes Kebede Gebremariam, Elin Kolle, Jakob Tarp

**Affiliations:** 1 Department of Sports Sciences, Norwegian School of Sport Sciences, PO Box 4014, Ullevaal stadion, 0806 Oslo, Norway; 2 Department of Community Medicine and Global Health, Institute of Health and Society, Faculty of Medicine, University of Oslo, Oslo, Norway; 3 Department of Clinical Epidemiology, Department of Clinical Medicine, Aarhus University and Aarhus University Hospital, Aarhus, Denmark

**Keywords:** Physical activity, Socioeconomic position, Built environment, Children, Adolescence

## Abstract

**Background:**

Physical activity behaviors among children and adolescents are socioeconomically patterned. Understanding if, and how, the built environment contributes to socioeconomic inequalities in physical activity and for whom built environments are most important, can lead to the identification of intervention entry points to reduce inequalities in physical activity.

**Objective:**

To summarize the existing evidence among children and adolescents on (a) whether the built environment mediates the association between socioeconomic position and physical activity and (b) whether socioeconomic position moderates the association between the built environment and physical activity.

**Methods:**

A systematic literature search was conducted using PubMed, Embase, PsycINFO and Web of Science. Two independent reviewers screened articles for eligibility, extracted information from included studies and assessed risk of bias with the Quality Assessment Tool for Observational Cohort and Cross-Sectional studies. We performed a narrative evidence synthesis considering the totality of the evidence and by study characteristics such as geographic region, age group, and exposure-outcome assessment methodology. The reporting was conducted in agreement with the Preferred Reporting Items for Systematic Reviews and Meta-Analysis (PRISMA) statement.

**Results:**

A total of 28 papers were included. In general, the studies were of low methodological quality. There was no evidence to support that the built environment functions as a mediator in the relationship between socioeconomic position and physical activity. We observed inconclusive moderation patterns with five studies reporting stronger associations between features of the built environment and physical activity among high socioeconomic positioned youths. Seven studies reported stronger associations among low socioeconomic positioned youth and fourteen studies reported no difference in associations. We observed different moderation patterns across geographical regions (Europe vs. US) indicating that, in Europe, having a walkable neighborhood is important for low socioeconomic positioned youth only. No differences in moderation patterns were observed for younger vs. older children or activity domains.

**Conclusion:**

Current evidence does not support a strong interplay between built environment and socioeconomic position on physical activity in youth. However, given the low quality of the evidence, firm conclusions cannot be made, and additional high-quality research is likely to have substantial impact on the evidence base.

**Supplementary Information:**

The online version contains supplementary material available at 10.1186/s12966-022-01385-y.

## Background

Physical inactivity is considered a global pandemic as most children and adolescents fail to meet the current recommendations of minimum 60 min of moderate-to-vigorous physical activity per day [1, 2]. This is of concern as low levels of physical activity among youth is associated with an increased risk of developing obesity, metabolic syndrome, poor mental health, and low quality of life [3–5]. Research evidence suggests that activity behaviors are socioeconomically patterned as children with low socioeconomic position (SEP) spend less time being physically active during leisure time and engage in less vigorous intensity activities, compared to their peers with high SEP [6, 7]. These domain- and intensity-specific differences are important as vigorous physical activity is considered to elicit stronger beneficial health effects compared with lower intensity physical activity [8].

Physical activity is a complex behavior, likely affected by determinants at multiple levels. According to ecological models, the built environment exerts a crucial influence on physical activity behaviors [9]. This is supported by several systematic reviews showing that people living in walkable, safe and greener neighborhoods tend to have higher levels of physical activity [10–16].

Socioeconomic position, at the individual- or the area-level, and the built environment are thought to be interrelated, and mediating and moderating pathways should be considered when these are related to health outcomes [17]. A mediator is defined as a variable that accounts for some or all of a given exposure-outcome association. On the other hand, a moderator is defined as a variable that affects the direction and/or strength of the relationship between the exposure and the outcome [18]. In other words, mediation says something about *why* two variables are related, while moderation says something about *when* two variables are related. According to the environmental justice model [19], there are two hypotheses describing the possible pathways of this relationship. The first hypothesis depicts that exposure to environmental burdens and benefits are unequally distributed across socioeconomic groups. This implies that children and adolescents with high SEP might be exposed to an environment that facilitates physical activity. Conversely, children and adolescents with low SEP might be exposed to an environment that impedes physical activity behavior. Thus, differences in the built environment can potentially account for some of the socioeconomic gradient in physical activity behavior and thereby act as a mediator in this relationship (the why). The second hypothesis states that SEP might influence the direction and/or strength of the relationship between the built environment and physical activity, thereby acting as a moderator in this relationship. For instance, children with low SEP may be more dependent on their neighborhood built environment for physical activity compared to their peers with high SEP. This could be due to the lack of financial and logistic resources to partake in organized sports that may happen outside their neighborhood (the when).

Understanding how the built environment may contribute to socioeconomic inequalities in physical activity among children and adolescents and for whom built environments are most important, can potentially lead to the identification of intervention entry points to reduce inequalities in physical activity [16]. Since interventions in the built environment can reach a vast number of people, these interventions can be particularly powerful, ultimately contributing to combating the physical inactivity pandemic and reducing inequalities in health.

Two systematic reviews have investigated the interrelationship between SEP, built environment and physical activity [20, 21]. However, only two studies including children and adolescents were identified by these reviews. Children and adolescents’ physical activity behaviors are more unstructured in comparison with adults, therefore, the dependency and engagement with the built environment may be greater [22, 23]. Thus, the findings from studies on adults cannot necessarily be generalized to children and adolescents.

The research output focusing on children and adolescents has increased rapidly in recent years [24–30]. Consequently, this systematic review aims to summarize the existing evidence among children and adolescents on (a) whether the built environment mediates the association between SEP and physical activity and (b) whether SEP moderates the association between the built environment and physical activity.

## Methods

### Search strategy

The search strategy was developed by all authors in collaboration with a librarian information specialist. The search terms and complementary key words were identified by a three-step process. First, a preliminary search was conducted in PubMed in June 2020 with the aim of identifying relevant articles with relevant keywords. Second, keywords from previous systematic reviews [13, 21, 31, 32] were reviewed and selected. Finally, expertise within the research group was solicited. Four electronic databases were searched; PubMed (listed as MEDLINE in the pre-specified protocol), Embase, PsycINFO and Web of Science from inception to the 4th of August 2021. The search matrix consisted of four key constructs covering physical activity, the built environment, SEP and age group (0–18 years old). The search was adapted to the relevant databases. The search matrix used in Pubmed, Embase, Web of Science and PsychINFO is available in additional file﻿ 1. The search was limited to articles written in English or a Scandinavian language. Grey literature was not consulted, and cross-references were not checked due to the comprehensive search strategy and large number of hits. Systematic search matrixes within equity and built environment research are challenging to develop because of the many terms used to describe key concepts [32]. Thus, we chose to develop a comprehensive search strategy without restrictions in order to maximize sensitivity. The review was registered in PROSPERO International Prospective Register of Systematic Reviews (number CRD42020184590). The reporting was conducted in agreement with the Preferred Reporting Items for Systematic Reviews and Meta-Analysis (PRISMA) statement [33]. The checklist is available in additional file﻿ 2.

To be eligible for review, the studies needed to focus on healthy children and adolescents between the ages of 0–18 years old. Studies focusing on specific clinical populations or studies with participants with a mean age exceeding 18 years old were excluded. All study designs except qualitative studies were eligible for inclusion (i.e. cross-sectional, retrospective, prospective, experimental or quasi-experimental). Studies were eligible if they included mediation or moderation analyses, or if they stratified analysis by SEP or built environment.

The main outcome, physical activity, could be measured with devices or be self-reported by questionnaires. We included total and domain-specific physical activity. This could be total physical activity, minutes spent in light intensity, moderate-to-vigorous intensity, leisure-time physical activity or active transportation. We did not include studies focusing on a single physical activity behavior, e.g. sports participation, as sports participation only accounts for a small proportion of total leisure time activities. The built environment could be measured objectively by Geographical Information Systems, field audits or virtual audits, or perceived, assessed through questionnaires. The measure had to focus on the built environment around the home and could include, but was not limited to, urban/rural comparison, walkability, land use-mix, access/proximity to recreational facilities, access/proximity to public open green spaces, aesthetics or safety from traffic. Studies using built environment around schools as a proxy of individual neighborhood environment were excluded. Studies focusing on social aspects of the environment (e.g. crime, drugs, fear of gangs etc.) were excluded. This also applied to studies using composite indexes including both social and built environment measures, without isolating the effect of the built environment. We included socioeconomic variables determined at the individual level (e.g. education, income, occupation, family affluence scale) and neighborhood level (e.g. deprivation index, median income of surrounding area).

### Screening and data extraction

Two reviewers (OA and JT) screened articles independently by title and abstract for inclusion. Disagreements were resolved by discussion. As mediation/moderation analyses often are secondary analyses which does not necessarily appear in the title or abstract, all studies with reference to either the built environment and physical activity or SEP and physical activity, passed the title/abstract screening. Finally, all full text articles were independently reviewed by the same two researchers for final eligibility assessment.

The following data were extracted from the included studies: authors, year of publication, year of study, journal, setting (country), study design, sample size, age, % girls, participation rate, length of follow-up (if applicable), exposure and outcome measurements and definitions, potential confounders (age, sex, BMI, parental self-selection, self-efficacy, social support, ethnicity (if relevant), sexual maturity (if relevant age group)), relevant test statistics and a summary of the findings. COVIDENCE was used for reference management [34].

Due to the heterogeneity in measurements and analytical methods applied, we determined that the data could not be meaningfully meta-analyzed. Thus, a narrative synthesis was conducted. Characteristics of the included studies were examined and summarized in tables and grouped based on whether the focus was on mediation or moderation effect. We coded studies as mediation models if it was possible to identify the isolated contribution of adjustment/adding indices of the built environment to the statistical model or if a formal test of mediation was conducted. This could be through specific reports of indirect effects or, through the difference in reported effect-sizes after adding variables representing features of the built environment to the statistical model. In studies using a formal mediation analysis, mediation was determined if the coefficient changed from significant to non-significant, otherwise a qualitative assessment of the change in coefficients after adjustment/adding indices of the built environment was conducted. We did not set a cut-off point required for minimum change in the coefficients. Moderation was established if a significant built environment x SEP interaction term was reported. If no formal test of interaction was conducted but studies reported effect sizes for built environment across socioeconomic strata (i.e. stratified analysis), we performed a qualitative assessment of variation in effect sizes to determine if the built environment was more important in specific subgroups. Finally, we stratified the results based on physical activity domain, perceived vs. objective determination of the built environment, type of SEP marker and geography, to explore heterogeneity among study results.

For ease of interpretation, we chose to group the built environment characteristics using an adapted version of the taxonomy of walking needs [35], which has also been used in a previous review [36]. The taxonomy classifies environmental factors in four key domains; accessibility, safety, comfort and pleasurability. We disaggregated accessibility into general accessibility (e.g. walkability, land-use mix, street connectivity, urban/rural measures) and access to recreational facilities as these features can influence physical activity behavior independently of each other and they are related to different physical activity domains [31]. We retained the term safety but limited the term to only include safety from traffic. The concept of pleasureability was retained, but we changed the term to aesthetics as it more accurately reflects the assessed features of the built environment.

### Risk of bias assessment

We used the Quality Assessment Tool for Observational Cohort and Cross-Sectional studies for risk of bias assessment [37]. The tool consists of 14 questions. We excluded question 1 and 2 as they refer to reporting rather than risk of bias. We further grouped the questions in four domains according to selection bias, information bias, confounding and temporality. A global rating of low risk of bias was given if all domains were rated with low risk. A global rating of some risk of bias was given if the study raised some concerns in at least one domain and had no domains reported as high risk of bias. Finally, a global rating of high risk of bias was given if the study had a high risk of bias in at least one domain or some risk of bias for multiple domains that substantially lowers confidence in the results. For more details on how the risk of bias assessment was conducted, please consult ﻿additional file 3.

## Results

**Fig. 1:**
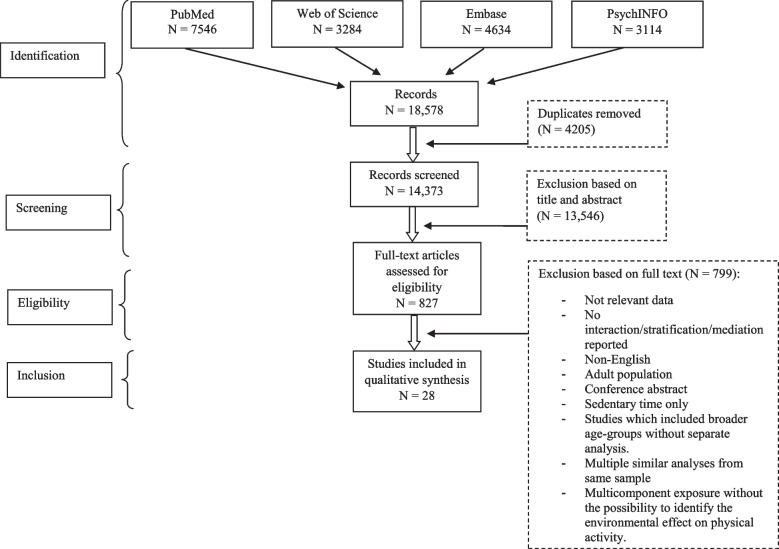
Flow chart of the literature search

The search returned 14,373 unique articles for the title and abstract screening. Of these, 13,546 papers were excluded, resulting in 827 full text articles to be screened. The final sample consisted of 28 papers that were eligible for review. Figure 1 provides further details. One eligible study was excluded [38] as the authors reported on the same sample in a follow-up study including additional adjustment for relevant confounding (27). There was 95% agreement for the title and abstract screening and 92% agreement for full text screening.

### Study characteristics

Study characteristics are presented in Tables 1 and 2. The included studies were published between 2006 and 2021. All the included studies, except one [26], had a cross-sectional design. Twelve studies were from North America, eleven studies were from Europe, two from South America, two from Africa, and one from Australia. Eleven studies focused on children [22, 26–28, 39–45], six on adolescents [23, 29, 46–49] and eleven studies included a mixed age group [24, 25, 30, 50–57]. The total study sample included 107,159 participants. All studies included a mixed-gender sample, with approximately 50% girls. The sample sizes varied from 259 [45] to 44,631 [54] participants.

**Table 1:** Study characteristics and summary of findings for mediation studies

Authors (year), study	Study setting & study design	N & age & % girls	Assessment of built enviornment	Assessment of socioeconomic position	Outcome	Additional variables included in the statistical model	Type of analysis	Findings
Kim 2020 [27] Geographic Research on Wellbeing	USA Cross-sectional	2493 Age range: 4 − 10 48.3% girls	GIS Access to recreational facilities	Parental reported family income	Parental reported Total physical activity	Age, sex, race/ethnicity, mother’s marital status, mother’s education, neighborhood SEP history	Mediation-model	No evidence of mediation. Distance to nearest park did not change the association between SEP and physical activity. →
Villanueva 2015 [57] Madrid City Health Survey	Spain Cross-sectional	727 Age range: 6–15 46% girls	GIS Accessibility Access to recreational facilities	Area-level unemployment rate and home surface area	Parental reported Total physical activity	Age, sex, parental education and professional qualifications	Mediation-model	No evidence of mediation. Availability of retail shops did not change the association between SEP and physical activity. Access to recreational facilities was not tested as a mediator. →

*SEP* Socioeconomic position, *SD S*tandard deviation, *GIS* Geographical information systems, → = no mediation

**Table 2:** Study characteristics and summary of findings for studies including moderation or stratified analysis

Authors (year), study	Study setting & study design	N & age & % girls	Assessment of built enviornment	Assessment of socioeconomic position	Outcome	Additional variables included in the statistical model	Type of analysis	Findings
Babey 2008 [50] California Health Interview Survey	USA Cross-sectional	4010 Age range: 12–17 % girls not stated	Perceived environment Access to recreational facilities	Parental-reported income	Self-reported Total physical activity	Age, sex, race/ethnicity, urbanicity, housing types, neighborhood safety	Formal test of interaction	No significant interaction. Models of regular activity and inactivity that tested interactions between access to a safe park and SEP were not significant. →
Bringolf-Isler 2014 [24] Pooled analysis of 7 Swiss studies	Switzerland Cross-sectional	1742 Age range: 4–17 498% girls	GIS Accessibility Aesthetics Safety	Area-level index	Accelerometery Total physical activity Moderate-to-vigorous physical activity	Age, sex, season, accelerometer model, study-cluster, + moderate-to-vigorous physical activity for wear-time	Formal test of interaction	Significant interaction for total physical activity and moderate-to-vigorous physical activity. Associations between physical activity and accessibility were stronger in low SEP neighborhoods. ↑
Bringolf-Isler 2019 [25] Swiss children’s Objectively measured Physical Activity (SPOHYA)	Switzerland Cross-sectional	1306 Age range: 6-16 9% girls	Perceived environment Safety Access to recreational facilities GIS Accessibility Safety Aesthetics	Area-level index	Accelerometery Moderate-to-vigorous physical activity	Age, sex, household income, language region, urbanicity, individual SEP	Formal test of interaction	Significant interaction for moderate-to-vigorous physical activity. Associations between physical activity and accessibility were positive in low SEP areas, but negative in high SEP areas. ↑
Clennin 2019 [26] Transitions and Activity Changes in Kids	USA. Prospective 2 year of follow-up	660 Mean (SD) age: 10.6 (0.1) 4% girls	GIS Access of recreational facilities	Area-level index.	Accelerometery Total physical activity	Age, sex, race/ethnicity, parent education, and weight-status	Formal test of interaction	No significant interaction. Access to recreational facilities did not moderate the association between deprivation and change in physical activity over two years. →
da Silva 2018 [55] Brazilian part of the International Physical Activity and Environment Network (IPEN) Study.	Brazil Cross-sectional	495 Age range: 12–17 years 1% girls	Perceived environment Safety	Parental-reported SEP Index	Self-reported Active transportation	Age, sex, perception of time spent to walk to school, leisure time moderate-to-vigorous physical activity, period spent in school + car ownership for analysis of parent reported built environment	Stratified analysis	Poisson regression showed largely similar weak associations with active transportation across SEP-categories for both adolescent and parent reported built environment. →
De Meester 2012 [23] Belgian Physical Activity study in Youth (BEPAS-Y)	Belgium Cross-sectional	513 Mean (SD) age: 14.6 (0.9). 0% girls	GIS Accessibility	Area-level household income	Accelerometry Total physical activity MVPA	Parental employment and educational attainment of mother and father, neighborhood-clustering	Formal test of interaction	Significant interaction for moderate-to-vigorous physical activity and total physical activity. Accessibility was positively associated with physical activity among children living in low SEP neighborhoods only. ↑
D’Haese 2014 [22] Belgian Physical Activity study in Children (BEPAS-Child)	Belgium Cross-sectional	494 Mean (SD) age: 10.1 (0.9) 55% girls	GIS Accessibility	Area-level household income	Self-reported Active transportation Accelerometry Moderate-to-vigorous physical activity	Age, sex, mother or father with college/university education	Formal test of interaction	Significant interaction for walking for transportation during leisure time. Accessibility was associated with more active transportation during leisure time among children living in low SEP neighborhoods only. No interaction for moderate-to-vigorous physical activity and active transportation to school was found. ↑
Diaz 2019 [46] No study name	Brazil Cross-sectional	1130 Mean (SD) age: 16.5 (1.1) 3% girls	Perceived environment Accessibility Safety Aesthetics GIS Accessibility	Self-report SEP index	Self-reported Active transportation	Age, sex, SEP, class cluster	Stratified analysis	The associations between thirteen different built environment exposures and active transportation were largely similar and non-significant across SEP tertiles. Perceived land-use mix and perceived access to recreational facilities were associated with active commuting to school among adolescents in middle SEP tertile only. →.
Hunter 2020 [39] Recording and Evaluating Activity in a Modified Park (REVAMP)	Australia Cross-sectional	1212 Mean (SD) age: 8.5 (3.6). 483% girls	Perceived environment Accessibility Access to recreational facilities	Area-level index	Parental reported Total physical activity	Sex, typical behaviour of child during last 7 days, park recruitment area, year of data-collection	Formal test of interaction	Significant interaction for total physical activity. Park quality was positively associated with physical activity among children living in high SEP neighborhoods, but not in low and medium SEP neighborhoods. ↓
Isgor 2011 [47] Child Development Supplement of the Panel Study of Income Dynamics Study	USA Cross-sectional	634 Mean (SD) age: 15.8 (1.22) 506% girls	GIS Access to recreational facilities	Self-reported family income	Self-report Total physical activity	Age, sex, ethnicity, marital status of family head, mother’s education, mother’s work status, zip-code level urbanization, zip-code level median household income	Stratified analysis.	No significant difference by SEP in the association between facility availability and PA among male adolescents. Statistically significant differences by SEP in the associations between facility availability and physical activity among female adolescents. Additional facilities being more important in low SEP but no impact in high SEP. ↑
Johanson 2012 [51] Health Behaviour in School-aged Children + Stockholm county	Sweden Cross-sectional	5423 5th − 9th grade 50% girls	Perceived environment Accessibility	Self-reported employment type	Self-report Active transportation	Car ownership	Stratified analysis.	In urban areas, children of low SEP were more likely to use active transportation compared with children of high SEP. In rural areas, only children of self-employed persons (including farmers) were less likely to use active commuting compared to those of intermediate- to high-level salaried employees. ↑
Kerr 2006 [52] Neighborhood Quality of Life Study	USA Cross-sectional	259 Mean (SD) age 11.3 (4) 486% girls	GIS Accessibility	Area-level household income	Parental reported Active transportation	Sex and age	Formal test of interaction	Significant interaction for active transportation. In low-walkability neighborhoods, income was not related to active transportation. In high-walkability neighborhoods, children in high-income neighborhoods were more likely to actively commute. ↓
Kim 2020 [53] Healthy Communities Study	USA Cross-sectional	4114 Age range: 4–15 50% girls	GIS Accessibility	Parental reported household income	Self-report Moderate-to-vigorous physical activity	Age, sex, race/ethnicity, parent education, neighborhood SEP history	Stratified analysis	No significant difference by SEP in the association between urbanicity and PA among children. →
Liu 2008 [54] National Survey of Children’s Health	USA Cross-sectional	44,631 Age range: 10-17 49% girls	GIS Accessibility	Parental reported family poverty status	Parental reported Total physical activity	Age, sex, race/ethnicity, overall health status, screen time, household poverty status/ parental education (mutual adjustment), number of children in household, parental physical activity, Perceived neighborhood safety	Stratified analysis	No consistent and meaningful difference by SEP was observed in the association between urbanicity and PA among children. →
McCrorie 2020 [28] Studying Physical Activity in Children’s Environments across Scotland	Scotland Cross-sectional	774 Mean (SD) age: 11.1 (0.3) 53% girls	GIS Accessibility	Household income	Accelerometry Total physical activity, Light intensity physical activity Moderate-to-vigorous physical activity	Sex, wear-time, number of valid days, season, distance to school, data zone walkability	Formal test of interaction	No significant interaction. The relationship between urban/rural settlement did not vary by household income quintile. →
Molina-Garcia 2017 [40] International Physical Activity and Environment Network (Spain)	Spain Cross-sectional	310 Mean (SD) age: 11.2 (0.5). 49% girls	GIS Accessibility	Parental reported education	Self-report Active transportation	Unclear as variables removed if p > 0.15. Considered variables are age, gender, weight-status, adult accompaniment to school, number of motor-vehicles per licensed driver, distance to school, environmental/safety and planning (psychosocial barriers, participant clustering within neighborhoods	Formal test of interaction	No significant interaction. Socioeconomic position did not influence the strength of association between the built environment and physical activity. →
Molina-Garcia 2017 [48] International Physical Activity and Environment Network (Spain)	Spain Cross-sectional	325 Mean (SD) age: 16.4 (0.8) 542% girls	GIS Accessibility	Parental reported education	Accelerometry Moderate-to-vigorous physical activity Self-report Active transportation	Unclear as variables removed if p > 0.15. Considered variables are age, gender, days per week living at primary address, distance to school, driver license, number of children < 18 years old living at household, number of motor vehicles per licensed driver, number of years living at current address, and workout equipment in or around home, participant clustering within neighborhoods	Formal test of interaction	Significant interaction for moderate-to-vigorous physical activity during weekends. In low-walkable neighborhoods, moderate-to-vigorous physical activity during weekends were higher with among low SEP children, while in high-walkable neighborhoods, moderate-to-vigorous physical activity during weekends were higher in high SEP. ↓
Oyeyemi 2014 [49] No study name	Nigeria Cross-sectional	1006 Mean (SD) age: 15.6 (1.7) 50% girls	Perceived environment Accessibility Access to recreational facilities Safety Aesthetics	Area-level income	Self-report Active transportation	Age, sex, school grade, parental education and occupation, all BE factors	Formal test of interaction	No significant interaction. Neighborhood income did not influence the associations of environmental perception and active transportation. Significant interaction is observed for leisure time in boys, however, as leisure time is restricted to sports participation, we have chosen to disregard this association. →
Page 2010 [41] Personal and Environmental Associations with Children’s Health	England Cross-sectional	1300 Age (range): 10-11 49% girls	Perceived environment Accessibility Safety Aesthetics	Area-level deprivation	Self-report Active transportation	Sex, daylight hours, pubertal stage, BMI, school clustering	Formal test of interaction	No significant interaction between neighborhood deprivation and accessibility in the association with active transportation (results not shown). →
Rodrigues 2018 [42] No study name	Portugal Cross-sectional	834 Mean (SD) age: 8.1 (1.2). 49% girls	GIS Accessibility	Parental reported education	Self-report Active transportation	Bivariate: none, Multivariable: not applied for relevant variables in boys( SEP not < 0.05 in bivariate model). Girls: age, sex, obesity, abdominal obesity, perceived safety, facilities close to home, distance to school, mother’s transportation, father’s transportation	Stratified analyses	No association between SEP and active transportation in urban setting. Bivariate analysis shows higher odds of active transportation with low SEP in rural setting. The association is attenuated after multivariable adjustment. →
Sallis 2018 [29] Teen Environment and Neighborhood Study	USA Cross-sectional	928 Mean (SD) age: 14.1 (1.4) 50.4% girls	GIS Accessibility	Area-level median household income	Accelerometery Moderate-to-vigorous physical activity Self-report Active transportation	Potential variables were (removed if p > 0.15): age, sex, race/ethnicity, has driver’s license, attends school away from home, days/week living at current address, works outside the home, number of children in household, number of motor vehicles per licensed driver, years at current address, walkability-related reasons for moving here, study site, census block clustering (+ for accelerometry: acc model and wear-time)	Formal test of interaction	Significant interaction for active transportation to school. In low walkable areas, low SEP children had higher prevalence of active transportation than high income children. No difference in active transportation between low and high SEP in high walkable areas. No significant interaction for moderate-to-vigorous physical activity. ↓
Shams-White 2021 [30] Family Life, Activity, Sun, Health and Eating Study	USA Cross-sectional	1295 Age range: 12-17 50.4% girls	GIS Accessibility	Area-level index	Self-report Moderate-to-vigorous physical activity	Age, gender, ethnicity, parent education, neighborhood urban/rural location	Formal test of interaction	Significant interaction for moderate-to-vigorous physical activity. Strongest association between built environment and physical activity in middle SEP. No association with moderate-to-vigorous physical activity in lowest and highest SEP (Q2-Q4 have slopes) →
Stone 2014 [43] Project BEAT	Canada Cross-sectional	856 Mean (SD) age: 11.0 (0.6). 55% girls	GIS Accessibility	Area-level median household income	Accelerometry Total physical activity Light intensity physical activity Moderate-to-vigorous physical activity	None	Stratified analysis	Main effect of SEP-built environment combination for weekday total, MVPA and LPA in boys and girls are presented. These are further stratified by independent mobility, precluding a clear interpretation of the results. →
Su 2013 [44] Southern California Children’s Health Study	USA Cross-sectional	4338 Mean (SD) age: 6.6 (0.7) 48.2% girls	GIS Accessibility	Parental reported education	Parental reported Active transportation	Age, sex, ethnicity, distance to school, traffic density, percent government and institutional, percent free and reduced-price meals program	Formal test of interaction	Significant interaction for active transportation. Accessibility associated with more frequent active transportation among high SEP children only. ↓
Uys 2016 [45] The International Study og Childhood Obesity, Lifestyle and the Environment (South Africa)	South Africa Cross-sectional	258 Mean (SD) age: 10.2 (0.6). 59% girls	GIS Access to recreational facilities Perceived environment Access to recreational facilities Safety	Self-reported household income	Accelerometery Total physical activity Moderate-to-vigorous physical activity	Age, sex	Formal test of interaction	Significant interaction for moderate-to-vigorous physical activity. Physical activity facilities positively associated with physical activity in low SEP children, but not in high SEP children. High traffic risk was associated with less activity among low SEP children, but not in high SEP children. ↑
Uzochukwu 2017 [56] National Survey of Children’s Health	USA Cross-sectional	25,092 Mean (SD) age: 13.6 range (10–17) 48% girls	GIS Accessibility Perceived environment Accessibility Aesthetics	Parental reported household income	Parental reported Total physical activity	Race/ethnicity, SEP, physical amenities, social cohesion, physical detractions, social detractions.	Stratified analysis Formal test of interaction	Stratified analysis suggested stronger correlation between SEP and physical activity in rural areas. No significant interaction. →

*SEP* Socioeconomic position, *SD* Standard deviation, *GIS* Geographical information systems, → = no interaction, ↑ = significant interaction in favor of low SEP, ↓ = significant interaction in favor of high SEP

Physical activity was most commonly measured by self-report [22, 27, 29, 30, 39–42, 44, 46–57], while ten studies measured physical activity with accelerometers [22–26, 28, 29, 43, 45, 48]. The built environment was most commonly assessed objectively by geographical information systems [22–30, 40, 42–48, 52–54, 56, 57]. Ten studies measured the perceived environment through various questionnaires [25, 39, 41, 45, 46, 49–51, 55, 56] and four studies measured both objective and perceived environment [25, 45, 46, 56]. Socioeconomic position was most frequently measured using a composite index [22–26, 30, 39, 41, 46, 55, 57], followed by income [27–29, 43, 45, 47, 49, 50, 52–54, 56], education [40, 42, 44, 48] and occupation [51]. Fourteen studies measured SEP at the area-level [22–26, 29, 30, 39, 41, 43, 49, 52, 57], while fifteen measured SEP at the individual level [27, 28, 40, 42, 44–48, 50, 51, 53–56]. More details are available in Tables 1 and 2.

### Mediation

Two studies [27, 57] investigated whether the built environment mediated the relationship between SEP and physical activity. Villanueva et al. reported change in coefficients without any formal test of mediation [57], while Kim et al. [27] conducted a formal test for mediation using the Baron and Kenny ﻿approach [18]. Neither of the studies found any evidence of mediation, as indicated by only minor or no change in reported odds ratios of the association between physical activity and SEP after adding indices of the built environment to the statistical model.

### Moderation

Twenty-six studies investigated whether the association between the built environment and physical activity varied across socioeconomic strata, either by examining formal SEP-by-built environment moderations [22–26, 28–30, 39–41, 44, 45, 48–50, 52, 56] or through stratified analysis [42, 46, 47, 51, 53–56]. Five studies [29, 39, 44, 48, 52] reported stronger associations between built environment and physical activity behaviors among youth with high SEP, while seven studies [22–25, 45, 47, 51] reported stronger associations among youth with low SEP. Fourteen studies reported no differences in the association between built environment and physical activity according to SEP strata [26, 28, 30, 40–43, 46, 49, 50, 53–56]. All studies that reported an association in either direction reported so for accessibility measures or access to recreational facilities. No moderation effect was found when indices of safety or aesthetics were used to characterize the built environment.

Stratifying the studies according to physical activity domain, perceived vs. objective determination of built environment, or type of SEP marker did not change the results. There were, however, differences according to geographical regions with contrasting findings for the US and Europe. In Europe, four of five studies [23–25, 51] focusing on adolescents or a mixed age-group reported the built environment to be most important among youth with low SEP, while one study [48] reported built environment to be most important in youth with high SEP. In the US, this relationship was inconclusive, with most studies showing no association [30, 50, 53, 54, 56] or opposite direction of what was observed in the European studies [29, 44, 52]. All studies that focused on active transportation reported the built environment to be most important among youth with high SEP. [29, 44, 52]. This was contradictory to two European studies [22, 51] which found the built environment to be more strongly associated with active transport in youth with low SEP. Unfortunately, there were too few studies in other geographical regions to draw any additional geographical patterns.

### Risk of bias

The risk of bias assessment is presented in detail in additional file 3. In summary, all studies were rated as having a high risk of bias, mainly due to the cross-sectional nature of the evidence and insufficient control of potential confounding factors. The lack of adjustment for parental self-selection bias was the main reason for receiving a high risk of bias in the confounding domain.

## Discussion

This systematic review summarized available literature describing the interplay between the built environment and SEP in determining physical activity in children and adolescents. There was no evidence to support that the built environment functions as a mediator in the relationship between SEP and physical activity. Conflicting evidence was found for SEP as a moderator in the association between the built environment and physical activity with different patterns across geographical regions. Included studies were cross-sectional with insufficient control for confounding. Current evidence does not support a strong interplay between built environment and SEP in determining physical activity in youth. However, given the quality of the evidence, firm conclusions cannot be made and additional high-quality research is needed.

### Mediation

Based on limited evidence, our results do not suggest that the built environment is a part of the causal pathway between SEP and physical activity. While this is contrary to the environmental justice theory [58], another recent systematic review supports our findings [59]. Jacobs and colleagues did not find socioeconomic variation in accessibility of the built environment, suggesting the number of facilities providing opportunities for physical activity is similar in neighborhoods with low and high SEP [59]. This is relevant as both of the included studies testing the mediation hypothesis based their assessment of the built environment on geographic information systems and used accessibility measures. Importantly, while accessibility may be similar, the maintenance of recreational facilities and green areas is often poorer in neighborhoods with low SEP compared to neighborhoods with high SEP [60]. The maintenance and quality of these features is reported by children and adolescents themselves to be important for their outdoor play [61, 62]. Therefore, future studies should consider including other features of the built environment, such as aesthetics, to further evaluate the mediation hypothesis. Only two mediation studies were identified by the present review which limits our ability to draw general conclusions. Finally, it is worth mentioning that we identified three studies [17, 44, 63] testing whether SEP mediates the relationship between the built environment and physical activity. However, since there is a limited theoretical rationale for why such a relationship would exist, these were not included in the present review.

### Moderation

Most of the included studies in the present review examined the moderating effect of SEP in the association between the built environment and physical activity. The results were conflicting with different patterns in Europe compared with studies conduction in the US.

In Europe, all but one [48] of the included studies reported that the built environment was most important among youth with low SEP [22–25]. For instance, a study conducted in Belgium found that living in more walkable neighborhoods was associated with an additional 7.4 min/day of moderate-to-vigorous physical activity among adolescents with low SEP [23]. This corresponds to a walkability effect of 24%, which is likely to have a substantial impact on individual and population health. Conversely, living in more walkable neighborhoods was only associated with an additional 0.6 min/day of moderate-to-vigorous physical activity among adolescents with high SEP. Adolescents with high SEP can partake in organized sports which is often associated with a membership fee. Furthermore, they may also have better access to motorized transportation. Consequently, their physical activity behavior might be less influenced by their immediate neighborhood compared to their peers with low SEP [6, 23, 64, 65]. Thus, having a neighborhood which is considered conducive for walking and outdoor play can be particularly important for adolescents with low SEP [66, 67].

In the US, all studies focusing on active transport found that living in a neighborhood with better accessibility was most important for children and adolescents with high SEP [29, 44, 52]. For instance, Sallis and colleagues [29] found that living in a more walkable neighborhood was associated with 1.6 more walking/biking trips per week among youth with high SEP, but only 0.6 more trips among youth with low SEP. This can reflect that children and adolescents with low SEP have less choice about their mode of transportation and are using active transportation by necessity. Thus, a walkable neighborhood might not elicit more active transportation among youth with low SEP as they are already active commuters to begin with [29, 63].

Although the results from the European and American studies appear contradictory, they could speak to differences in urban designs between the continents. For instance, only European studies found walkability to be associated with higher physical activity among adolescents with low SEP. High walkability may reflect ease of access to destinations, but it may also reflect high traffic loads. This was demonstrated by an American study, which found greater land use mix (a component of walkability) to discourage physical activity [44]. Thus, it could be that walkability to a larger extent reflects high traffic loads in the US, and ease of access in Europe. Furthermore, there might be different cultures for physical activity behavior between the US and Europe. None of the five European studies focusing on active transportation found the built environment to be most important among youth with high SEP [22, 40–42, 51]. Compared to the US, active transportation is much more widespread in Europe. This is particularly true for Central- and North European countries [1, 68, 69] where cities are thought to be more walking- and cycling-friendly [70]. Thus, socioeconomic differences in active transportation may be smaller in these countries.

### Methodological considerations

Due to weak quality of the evidence, the results must be interpreted with caution. Mediation and moderation analyses derived from cross-sectional studies are only able to establish a relationship between three variables within a pre-specified framework, but it is not possible to determine whether this framework represents a causal process [71]. Thus, the cross-sectional design in all but one study [26] precluded determination of the temporal ordering of exposure and outcome, and limits inference about causality. Furthermore, all of the studies were rated as having high risk of bias, mainly due to inadequate control for confounding factors, and particularly parental self-selection bias was rarely addressed. Self-selection refers to the fact that people usually move to a neighborhood based on their preferences or lack of alternatives due to economic constraints [72]. Thus, an active person may choose to live in a neighborhood that facilitates physical activity. This is especially true for individuals with high SEP which have greater financial means to select their place of residence. Conversely, individuals with low SEP may be forced to select their neighborhood based on affordability. This can result in an overestimation of the impact of the built environment as those who choose to live in neighborhoods conducive for physical activity are likely to be active to begin with [73]. Although children and adolescents are unlikely to choose their neighborhood, parental self-selection should be adjusted for as parents are likely to influence their child’s activity behaviors [74]. For instance, if the parent’s main reason for moving to a neighborhood is having proximity to school and traffic safety, then the neighborhood effect on active transportation will be overestimated because this also supports the parents preferred behavior of the child, namely that they should walk to school [72, 75]. Importantly, active transportation was the physical activity outcome of interest in fourteen of the studies included in this review. We suspect parental self-selection is a major source of bias in these analyses. We therefore encourage future studies to collect and use information on neighborhood preferences in their statistical models.

The included studies used both objective and perceived built environment measures. The agreement between these measurement modalities are generally low, possibly because perception is influenced by individual personality characteristics [76, 77]. Furthermore, both administratively defined boundaries and egocentric defined boundaries were used to determine neighborhood exposure, neither of which perfectly reflects the actual neighborhood that children and adolescents themselves report using [78]. This likely leads to an underestimation of associations between the built environment and physical activity by virtue of introducing non-differential misclassification and thereby also potentially attenuating the moderating influence of SEP [79].

Built environment measures includes several aspects (e.g. accessibility, access to recreational facilities, safety and aesthetics) which are related to different physical activity domains. For instance, walkability may be more strongly related to active transport, while the presence of recreational facilities might be more strongly associated with leisure time physical activity [22]. Consequently, the conceptual matching of built environment features and the physical activity domains under analysis is important [80]. Several of the included studies [25, 26, 45, 47, 50] reported on the number of recreational facilities and green space without measuring leisure time physical activity. Importantly, the socioeconomic gradient is likely to be more pronounced for leisure-time physical activity compared to total physical activity [6, 7]. Thus, the lack of conceptual matching may have contributed to the inconsistent results.

Most studies conducting analyses of moderation did so in secondary analyses, without reporting power calculations. It is therefore reasonable to assume that several of the included studies were designed to detect main effects, not considering the sample size needed to detect potential differences in main effects across socioeconomic and built environment strata. Several of the studies reporting significant interactions reported very large effect sizes. For instance, a Belgian [23] and a Spanish [48] study found effects sizes to correspond to 7–10 min in moderate-to-vigorous physical activity, or 15–30% difference. It is likely that smaller, but meaningful, moderating effects could have been observed if the studies were designed with sufficient power to detect these differences. We would therefore encourage researchers to design studies considering the power needed to detect moderating effects and state the magnitude of an important effect.

Moderation was typically examined using binary categories with a median split [22, 24, 25, 29, 40, 43, 45, 50, 55]. This can make moderation difficult to observe, as difference between high and low SEP categories may be diluted. Interestingly, three studies omitted either the 5th, or the 5th and the 6th decile in their binary SEP categories, and all three reported a significant moderating effect of SEP [23, 48, 52]. This could suggest greater SEP contrasts are needed to identify associations. Consequently, future studies may consider securing greater heterogeneity between SEP groups.

Finally, one study which measured SEP-by-built environment interactions used individual SEP as exposure and then adjusted for area SEP [47], while others used area SEP and then adjusted for individual SEP [30, 49, 57]. The rationale for these approaches were not presented and we encourage researchers to state their logic behind attempting to isolate specific parts of SEP by adjusting for other indicators of SEP.

### Practical implications

The dearth of prospective and experimental studies represents a significant opportunity for the research community to move the science forward. Although randomized controlled trials in the built environment are extremely difficult to conduct, natural experiments are feasible and crucial to gain insight into the built environments impact on socioeconomic differences in children and adolescents physical activity behaviors. To achieve this, closer collaborations between researchers, policy makers and urban planners are needed. Despite the shortcomings of the present evidence, targeting pedestrian infrastructure to make neighborhoods more walkable looks to be a promising strategy to increase activity levels among adolescents with low SEP in Europe. Furthermore, the included studies from the US suggest targeting traffic safety measures in neighborhoods with low SEP is important to ensure safe routes to schools and for recreational purposes. Although such interventions in the built environment are costly, previous research has suggested that the cost-benefit is comparable to other health enhancing measures [21, 81]. Additionally, studies focusing on aesthetics were largely underrepresented in the present review. We would therefore encourage future studies to consider this aspect of the built environment as neighborhoods with high and low SEP may be equally rich in amenities, but they may vary greatly in their quality (e.g. run down/dirty parks and recreational facilities) [60]. Finally, there is a need for more studies from outside Europe and the US.

### Strength and limitations

The comprehensive search strategy and the utilization of two reviewers in all stages of the screening is a major strength of the present review as it minimized the chances of missing relevant articles. Furthermore, we aimed to maximize transparency by preregistering the review in PROSPERO and following the PRISMA 2020 guidelines [33].

The present review has some weaknesses that need to be addressed. We used vote counting as a method for synthesizing evidence, which does not account for the quality of studies, sample size or the effect size. However, the heterogeneity in measurements and analytical approaches precluded meta-analysis. We based our judgement of mediation and moderation on formal statistical examinations if available. However, some studies reported effect-sizes for the built environment across strata of SEP, allowing only for a qualitative comparison of effect-sizes across these strata, not a formal test of difference-in-difference. We did not apply a fixed criterion for these evaluations as we consider no specific meaningful threshold could be applied. Because we only included studies written in English or a Scandinavian language, there is a potential risk for selection bias. Furthermore, we chose to only include studies that measured a complete activity domain which meant that studies focusing on sports participation were excluded. This is unlikely to be a major issue as organized sports often happens outside of the immediate neighborhood. Finally, we did not include studies using built environment around schools as a proxy of individual neighborhood environment. Children and adolescents often live more than 1 km from their school [82, 83]. Thus, we did not consider school neighborhood to be a good proxy for individual neighborhood exposure.

## Conclusion

We found no evidence to support that the built environment functions as a mediator in the relationship between SEP and physical activity. Evidence from studies examining how SEP moderates the association between built environment and physical activity was inconclusive with some suggestions towards geographical differences. Targeting pedestrian infrastructure to make neighborhoods more walkable looks to be a promising strategy to increase activity levels among adolescents with low SEP in Europe, only. However, the current body of literature consists largely of low-quality evidence derived from cross-sectional studies. Thus, firm conclusions cannot be made. Closer collaborations between researchers, policy makers and urban planners is needed to design high quality research to determine role of the built environment in combating social inequalities in physical activity among children and adolescents.

## Supplementary Information

**﻿Additional file 1**.

**﻿Additiona﻿l file 2**.

**﻿Additional file 3**.

## Data Availability

All data generated/or analyzed during this study are included in this published article and its supplementary information files.

## Abbreviations

SEP: Socioeconomic position
