# Supplementary material for: Socioeconomic position, built environment and physical activity among children and adolescents: a systematic review of mediating and moderating effects
Source: Int J Behav Nutr Phys Act. 2022 Dec 12;19:149. doi: 10.1186/s12966-022-01385-y (PMC9743748; doi:10.1186/s12966-022-01385-y)
Supplement: Supplementary file 1 — ﻿Additional file 1. [file 12966_2022_1385_MOESM1_ESM.docx]

| **Additional file 1.  Search strategy and keywords** **used in Embase, Web of Science and PsychINFO.** | |
| --- | --- |
| **Set** | **Search terms** |
| #1 | (socioeconomic* or "socio-economic*" or "socio economic*" or equit* or affluen* or "high income" or "low income" or income* or occupation* or "education level*" or education* or poverty or injustice or deprivation* or "social determinant*" or "social class*" or "social status" or "occupational status") |
| #2 | ("physical* activ*" or "active travel*" or "active transport*" or "active commut*" or "transport mode*" or "transportation mode*" or "travel mode*" or exercise* or cycle or cycling or bicycle* or bicycling or cyclist* or walking or walk* or "physical* inactiv*" or sedentary) |
| #3 | ("built environment*" or "physical environment*" or "urban environment*" or "suburban environment*" or "community environment*" or "travel environment*" or "street environment*" or "obesogenic environment*" or accessibilit* or "population densit*" or "land use mix" or proximit* or "road environment*" or "urban design*" or "environmental design*" or "environmental characteristic*" or "green space*" or walkability or "open space*" or parks or facilities or neighborhood* or neighbourhood* or "objective environment*" or "perceived environment*" or "geographical information system*" or "spatial access" or "recreational facilit*" or "sports facilit*" or "street connectivity*" or "residential densit*" or "city planning" or "environmental design*" or "public transit" or "public transport*" or streetscape* or "traffic calm*" or sidewalk* or footpath* or infrastructure* or "fitness facilit*" or "public space*") |
| #4 | (adolescent* or adolescence or children* or youth* or child or student* or teen* or childhood) |
| Final search | #1 AND #2 AND #3 AND #4 |

| **Search strategy and keywords** **used in PubMed.** | |
| --- | --- |
| **Set** | **Search terms** |
| #1 | (socioeconomic or "socio-economic*" or "socio economic" OR "socio economics" or equity or equities or affluen* or "high income" or "low income" or income or occupation or occupations or "education level*" or education or poverty or injustice or deprivation or "social determinant*" or "social class*" or "social status" or "occupational status") |
| #2 | ("physical activity" or "active travel*" or "active transport*" or "active commut*" or "transport mode*" or "transportation mode*" or "travel mode*" or exercise or cycle or cycling or bicycle or bicycling or cyclist* or walking or walk* or "physical inactivity" or sedentary) |
| #3 | ("built environment*" or "physical environment*" or "urban environment*" or "suburban environment*" or "community environment*" or "travel environment" or "street environment*" or "obesogenic environment*" or accessibility or "population densit*" or "land use mix" or proximity or "road environment*" or "urban design*" or "environmental design*" or "environmental characteristic" or "environmental characteristics" or "green space*" or walkability or "open space*" or parks or facilities or neighborhood or neighbourhood or "objective environment*" or "perceived environment*" or "geographical information system*" or "spatial access" or "recreational facilit*" or "sports facilit*" or "street connectivity*" or "residential densit*" or "city planning" or "environmental design*" or "public transit" or "public transport*" or streetscape* or "traffic calm*" or sidewalk* or footpath* or infrastructure or "fitness facilit*" or "public space*") |
| #4 | (adolescent or adolescence or children or youth or child or student* or teen or childhood) |
| Final search | #1 AND #2 AND #3 AND #4 |
