# Supplementary material for: Socioeconomic position, built environment and physical activity among children and adolescents: a systematic review of mediating and moderating effects
Source: Int J Behav Nutr Phys Act. 2022 Dec 12;19:149. doi: 10.1186/s12966-022-01385-y (PMC9743748; doi:10.1186/s12966-022-01385-y)
Supplement: Supplementary file 3 — ﻿Additional file 3. [file 12966_2022_1385_MOESM3_ESM.docx]

|  | Selection | Information | Confounding | Temporality | Overall |
| --- | --- | --- | --- | --- | --- |
| **Mediation papers** |  |  |  |  |  |
| Kim 2020 |  |  |  |  |  |
| Villanueva 2015 |  |  |  |  |  |
| **Moderation papers** |  |  |  |  |  |
| Babey 2008 |  |  |  |  |  |
| Bringolf-Isler 2014 |  |  |  |  |  |
| Bringolf-Isler 2019 |  |  |  |  |  |
| Clennin 2019 |  |  |  |  |  |
| da Silva 2018 |  |  |  |  |  |
| De Meester 2012 |  |  |  |  |  |
| D’Haese 2014 |  |  |  |  |  |
| Diaz 2019 |  |  |  |  |  |
| Hunter 2020 |  |  |  |  |  |
| Isgor 2011 |  |  |  |  |  |
| Johanson 2012 |  |  |  |  |  |
| Kerr 2006 |  |  |  |  |  |
| Kim 2020 |  |  |  |  |  |
| Liu 2008 |  |  |  |  |  |
| McCrorie 2020 |  |  |  |  |  |
| Molina-Garcia 2017_a_ |  |  |  |  |  |
| Molina-Garcia 2017_b_ |  |  |  |  |  |
| Oyeyemi 2014 |  |  |  |  |  |
| Page 2010 |  |  |  |  |  |
| Rodrigues 2018 |  |  |  |  |  |
| Sallis 2018 |  |  |  |  |  |
| Shams-White 2021 |  |  |  |  |  |
| Stone 2014 |  |  |  |  |  |
| Su 2013* |  |  |  |  |  |
| Uys 2016 |  |  |  |  |  |
| Uzochukwu 2017 |  |  |  |  |  |
| = Low risk of bias = Some risk of bias = High risk of bias | | | | | |
|  | | | | | |

**Additional file 3.**

**Risk of bias assessment of the included studies conducted with the Quality Assessment Tool for Observational Cohort and Cross-Sectional Studies.**

**Guide for risk of bias assessment**

For the original tool and detailed instructions, please see: <https://www.nhlbi.nih.gov/health-topics/study-quality-assessment-tools>.

**SELECTION BIAS**

1. Was the participation rate of eligible persons at least 50 %?

*Note: Participation rate calculated as participants analyzed/eligible population * 100, where eligible population is defined as participants invited to participate in the study.*

1. Were all the subjects selected or recruited from the same or similar populations (including the same time period)? Were inclusion and exclusion criteria for being in the study prespecified and applied uniformly to all participants?

*Note: Yes = all subjects were recruited from the same community or a national representative sample. Inclusion and exclusion criteria for all participants must be clearly stated.*

1. Was loss to follow-up after baseline 20 % or less?

*Note: The proportion of subjects with baseline data who remained through the end of the study interval or had measures from more than one time-point.*

**INFORMATION BIAS**

1. For exposures that can vary in amount or level, did the study examine different levels of the exposure as related to the outcome (e.g., categories of exposure, or exposure measured as continuous variable)?

*Note: Formal interaction with two or more groups OR with linear combination (by including built environment x SEP interaction term). Built environment and SEP divided into two categories without formal test of interaction = poor.*

1. Were the exposure measures (independent variables) clearly defined, valid, reliable, and implemented consistently across all study participants?

*Note: Studies that used instruments or questionnaires without reporting or referring to sources on the validity/reliability of the measurement = poor. This applies for both SEP and built environment measures. For instance, neighborhood affluence scale, parental income or education = good, number of cars = poor. Tools/questionnaires used to measure the built environment should be derived from existing validated instruments/questionnaires = good.*

1. Were the outcome measures (dependent variables) clearly defined, valid, reliable, and implemented consistently across all study participants?

*Note: IPAQ, IPAQ Short, accelerometry = good. Studies that used very crude measurements of physical activity or questionnaires without reporting on the validity/reliability of the measurement = No. E.g. parental reported physical activity/sports less than once a month vs. one or more times a month in his/her free time = poor*

1. Was a sample size justification, power description, or variance and effect estimates provided?

*Note: Was this provided for the specific mediation/moderation analysis? If not = No*

1. Were the outcome assessors blinded to the exposure status of the participants?

*Note: Device-measured physical activity = yes. Questionnaire = no.*

**CONFOUNDING**

1. Were key potential confounding variables measured and adjusted statistically for their impact on the relationship between exposure(s) and outcome(s)?

*Note: If adjusted for age, sex, BMI, parents’ reason for moving to the neighborhood (or related indicators e.g. parental physical activity) and ethnicity (if relevant) = yes*

**TEMPORALITY**

1. For the analyses in this paper, were the exposure(s) of interest measured prior to the outcome(s) being measured?

*Note: Cross-sectional study = no, longitudinal study = yes*

1. Was the exposure(s) assessed more than once over time?

*Note: More than one time-point included.*

1. Was the timeframe sufficient so that one could reasonably expect to see an association between exposure and outcome if it existed?

*Note: Yes, if timeframe = ≥ 6 months*

**SCORING PROCEDURE**

| **Overall risk-of-bias judgment** | **Criteria** |
| --- | --- |
| **Low risk of bias** | The study is judged to be at low risk of bias for all domains for this result. |
| **Some risk of bias** | The study judged to raise some concerns in at least one domain for this result, but not to be high risk of bias for any domain. |
| **High risk of bias** | The study is judged to be at high risk of bias in at least one domain for this result OR The study is judged to have some concerns for multiple domains in a way that substantially lowers confidence in the result. |
